# Left atrial strain predicts fibrosis of left atrial appendage in patients with atrial fibrillation undergoing totally thoracoscopic ablation

**DOI:** 10.3389/fcvm.2023.1130372

**Published:** 2023-05-17

**Authors:** Jihoon Kim, Sung-Ji Park, Dong Seop Jeong, Suryeun Chung, Kina Jeon, Minjung Bak, Darae Kim, Eun Kyoung Kim, Sung-A Chang, Sang-Chol Lee, Seung Woo Park

**Affiliations:** ^1^ Division of Cardiology, Department of Medicine, Heart Vascular Stroke Institute, Samsung Medical Center, Sungkyunkwan University School of Medicine, Seoul, Republic of Korea; ^2^ Department of Thoracic and Cardiovascular Surgery, Cardiac and Vascular Center, Samsung Medical Center, Sungkyunkwan University School of Medicine, Seoul, Republic of Korea

**Keywords:** atrial fibrillation, ablation, fibrosis, strain, prognosis

## Abstract

**Background:**

Left atrial (LA) fibrosis is related with development and severity of atrial fibrillation (AF). The aim of this study was to investigate the association between LA strain and LA fibrosis in patients undergoing totally thoracoscopic ablation (TTA) for AF.

**Methods:**

Between February 2012 and March 2015, a total of 128 patients who underwent TTA were enrolled from a tertiary hospital. Left atrial appendage (LAA) was harvested during surgery to determine the degree of fibrosis. LAA fibrosis was classified as mild (1st quartile), moderate (2nd and 3rd quartile), or severe (4th quartile). Clinical outcome was 5-year recurrence rate of AF detected on electrocardiogram or 24 h Holter monitoring.

**Results:**

The mean age was 54.3 ± 8.8 years and 18.8% had paroxysmal AF. Patients with mild LAA fibrosis had a significantly lower rate of recurrent AF (23.3%) at 5 years after TTA compared with those with moderate (51.4%; hazard ratio [HR] 2.69; 95% confidence interval [CI] 1.19–6.12) or severe (53.2%; HR 2.84; 95% CI 1.16–6.97) fibrosis. Among clinical and echocardiographic parameters, peak LA strain was the only predictor of mild LAA fibrosis (coefficient 0.10, *p* = 0.005) with the best cutoff value of 14.7% (area under the curve 0.732). The prevalence of mild LAA fibrosis was 40.6% in patients with peak LA strain ≥14.7%, but only 6.8% in those with peak LA strain <14.7%.

**Conclusions:**

In patients undergoing TTA for AF, mild LAA fibrosis was associated with a lower risk of 5-year AF recurrence. LA strain was the only predictor of mild LAA fibrosis that reflects a lower risk of 5-year AF recurrence.

## Introduction

Atrial fibrillation (AF) is the most common arrhythmia and a growing disease affecting more than 3 million new cases each year (1). Structural atrial remodeling is a key element in AF development and progression. There are number of studies demonstrating the relationship between atrial fibrosis and AF (2, 3). Atrial fibrosis can be directly approached by invasive methods. Endomyocardial biopsy (4) or surgical biopsy during open heart surgery (5–7) allows pathological evaluation of atrial fibrosis. Electroanatomic mapping also can be used to detect areas of remodeling or fibrosis (8). As a noninvasive method, delayed enhancement magnetic resonance imaging (MRI) provides information on left atrial (LA) fibrosis and predicts outcome after catheter ablation of AF (9, 10).

Traditionally, echocardiographic assessment of LA remodeling has been limited to LA diameter and volume, or tissue velocity. LA strain by two-dimensional speckle-tracking echocardiography enables to assess myocardial function and cardiac chamber deformation. In patients with AF, LA strain can predict successful electrical cardioversion (11) or catheter ablation (12). Although several studies have attempted to identify the direct relationship between LA strain and extent of fibrosis, these studies were conducted in patients with mitral valve disease (13) or undergoing heart transplantation (14), not those with AF. The aim of this study was to evaluate the association of LA strain with LA fibrosis and outcomes in patients with AF undergoing thoracoscopic ablation.

## Materials and methods

### Study population

Between February 2012 and March 2015, patients undergoing totally thoracoscopic ablation (TTA) were enrolled in a prospective registry in Samsung Medical Center, Republic of Korea. Patients with AF refractory to at least 1 antiarrhythmic drug or electrical cardioversion were candidates for TTA. The indications for TTA also included refractory symptom, history of stroke, or intolerance for anticoagulant therapy. TTA was contraindicated in patients with LA thrombi or who were intolerant of one-lung ventilation (15, 16).

**Figure 1:**
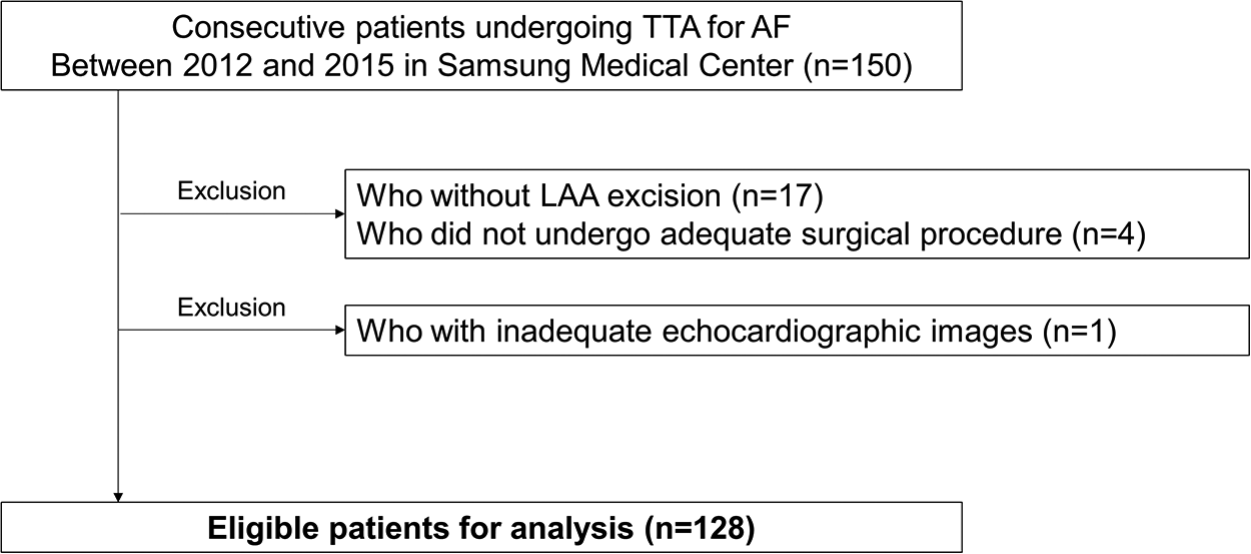
Study population. AF, atrial fibrillation; LAA, left atrial appendage; TTA, totally thoracoscopic ablation.

Among a total of 150 consecutive patients who underwent TTA during the study period, 22 patients were excluded from the present study as follows: (1) those who did not undergo adequate surgical procedure due to huge LA or severe pericardial adhesion (3 patients), or who underwent left atrial appendage (LAA) resection alone for stroke prevention (1 patient); (2) those who did not undergo LAA resection during the surgery (17 patients); (3) who did not have adequate echocardiographic images for analysis. Finally, 128 patients were eligible for final analysis (Figure 1). Eligible patients were divided into 3 groups according to the interquartile range of degree of LAA fibrosis as mild (1st quartile), moderate (2nd and 3rd quartile), or severe (4th quartile).

The Samsung Medical Center Institutional Review Bord approved the present study (2020-05-146-003).

### Thoracoscopic procedures

TTA was a video-assisted thoracoscopic surgical technique without thoracotomy and cardiopulmonary bypass. A bilateral approach was required, and the detailed techniques of TTA were described in our previous report (17). Ablation lines for pulmonary vein isolation were created using an AtriCure Isolator Transpolar Clamp (Atricure, Inc., Cincinnati, Ohio) and the left atrial roof and floor lesions connecting both pulmonary veins were created with a linear pen device (Atricure, Inc., Cincinnati, Ohio). After creating pulmonary vein isolation and box lesions, exit and entrance block test using an AtriCure Cooltip pen (Atricure, Inc., Cincinnati, Ohio) are performed. Next, the ganglionated plexuses were checked and ablated. The ligament of Marshall, which might be a source of adrenergic atrial tachycardia, was always divided and ablated (18). LAA was removed by stapling with an Echelon Flex 60 articulating endoscopic linear stapler (Ethicon Endo-Surgery Inc., Cincinnati, OH, USA).

### Postoperative care and endpoint

Patients were monitored in intensive care unit for the first 24 h. Heparin was administered after 2 h postoperatively and conversion to either warfarin or novel oral anticoagulant as soon as possible. Oral amiodarone was prescribed if the heart rate was >80 beats/min with AF rhythm at rest. Patients were followed up at 3, 6, and 12 months, and annually thereafter with 24 h Holter monitoring. Antiarrhythmic drugs were discontinued after 3 months or up to 6 months based on the results of 24 h Holter monitoring. Anticoagulants were also discontinued after 3 months considering the risk of stroke for each patient.

Clinical outcome was recurrent AF or atrial flutter (AFL) detected on electrocardiogram or lasting more than 30 s in 24 h Holter monitoring during the 5 years after TTA. The median follow-up duration of study subjects was 5.0 years.

### Histological analysis

**Figure 2:**
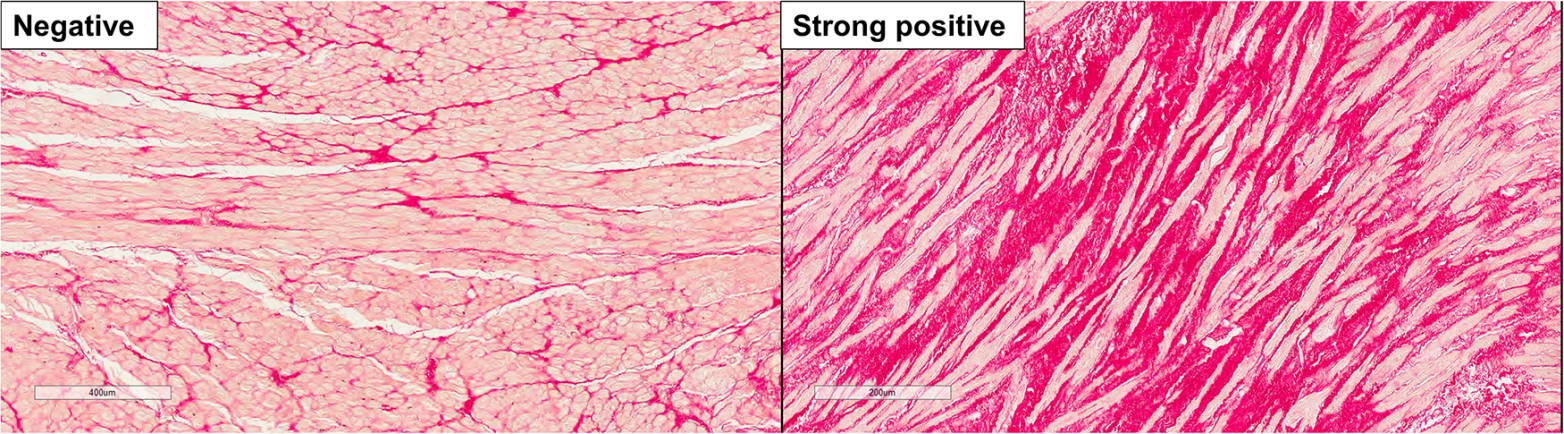
Representative histologic images of **left** atrial appendage fibrosis. **Left** panel, negative staining for fibrosis; **right** panel, strong positive staining for fibrosis.

We analyzed the pathology of LAA after resection. Portion of each LA tissue (about 2 × 4 mm to 4 × 10 mm) was fixed in 10% buffered formalin and embedded in paraffin (Figure 2). To quantify the extent of atrial fibrosis, sections (5 µm) stained with Picro Sirius red were scanned (×400) and the percentage of fibrosis area was automatically measured using the Image Pro Plus program (Media Cybernetics, Inc., Bethesda, MD). Because automatic measure of myocardial fibrosis tends to overestimate the real pathologic sclerotic tissue, we excluded non-pathologic fibrosis areas such as peri-adventitial, adipose tissue, and tangentially cut subendocardial portions. Average values of 5 random fields from each slide were calculated (19).

### Echocardiography and speckle-tracking imaging

Preoperative comprehensive transthoracic echocardiography was performed with commercially available equipment (Vivid 7, GE Medical Systems, Milwaukee, WI, USA; Acuson 512, Siemens Medical Solution, Mountain View, CA, USA, or; Sonos 5500, Philips Medical System, Andover, MA, USA) according to the practice guideline (20). Left ventricular end-diastolic and end-systolic diameter, and LA diameter were calculated from parasternal long-axis view. Left ventricular ejection fraction were calculated from 2-dimensional recordings using the modified biplane Simpson's method. LA volume was assessed by the modified biplane area-length method and was indexed to body surface area (LA volume index, LAVI). Early diastolic mitral inflow velocity (*E*) was measured using the pulsed wave Doppler method, by placing the sample volume at the level of the mitral valve leaflet tips. The tissue Doppler-derived early diastolic mitral annular velocity (*e*′) was measured from the septal corner of the mitral annulus in the apical 4-chamber view. For patients with AF rhythm, the average of 5 consecutive doppler signals was used.

Peak longitudinal LA strain (reservoir strain) was calculated using vender-independent dedicated software (2D cardiac Performance Analysis 1.4, Tomtec Imaging System) according to the current guideline (Supplementary Figure S1) (21). LA endocardial border was automatically traced by the software then was manually adjusted in both apical four- and two-chamber views. Pulmonary veins and LAA orifices were carefully excluded. The regions of interest encompassed endocardial border of the mitral annulus, and the thickness of regions of interest was adjusted to the thinnest part to adapt to the atria. The data were analyzed by two independent echocardiologists blinded to clinical status. All strain values were calculated from cardiac cycle with heart rate less than 110 per minute to avoid inadequate LA emptying or filling. LA stiffness index was defined as *E*/*e*′ divided by peak longitudinal LA strain.

### Statistical analysis

Continuous variables were presented as mean ± standard deviation or median [interquartile range]. Categorical variables were compared using the chi-square test. Continuous variables were compared using the Student's *t* test or Mann–Whitney *U* test as appropriate. Distribution of patients with mild, moderate, and severe LAA fibrosis was assessed according to the median value of LA strain, LA diameter and LAVI, paroxysmal AF, or LA stiffness index in baseline characteristics of the overall population. The incidence of recurrent AF or AFL at 5 years after TTA were estimated using the Kaplan-Meier method. Survival curves were compared with the log-rank test. The best cutoff value of extent of LAA fibrosis to maximize the difference in 5-year recurrence rate was estimated by plotting the standardized log-rank statistic. Association of LAA fibrosis extent with clinical or echocardiographic parameters was analyzed using logistic regression. In multivariable logistic regression analysis, strain-derived indices (LA strain and stiffness index) were not adjusted simultaneously because of multicollinearity. Receiver-operating characteristic analysis was used to find the best cutoff value of echocardiographic parameters for predicting mild LAA fibrosis.

Since the rhythm status during echocardiography may affect LA strain measurements, we conducted a subgroup analysis that included only patients with non-paroxysmal AF. All tests were two-sided, and a *p* value <0.05 was considered statistically significant. Statistical analysis was performed using R 3.6.2 (R Foundation for Statistical Computing).

## Results

### Baseline characteristics

Of 128 study subjects, mean age was 54.3 ± 8.8 years, 95.3% were male, and 18.8% had paroxysmal AF (Table 1). The median LA diameter and LAVI was 45.0 mm (40.0‒50.0) and 45.2 ml/m^2^ [36.4‒54.8], respectively. A median value of LA strain was 15.3% [12.1‒19.2] and a median value of LAA fibrosis extent was 38.6% [33.1‒44.7].

**Table 1:** Baseline characteristics.

Variables	Overall (*n* = 128)	Mild fibrosis (<33.1%, *n* = 32)	Moderate fibrosis (33.1%–44.7%, *n* = 64)	Severe fibrosis (≥44.7% *n* = 32)	*p* value
LAA fibrosis*, %	38.6 [33.1–44.7]	29.5 [27.5–31.3]	38.6 [36.1–41.0]	52.6 [47.6–56.2]	<0.001
**Clinical**					
Age, years	54.3 ± 8.8	54.5 ± 9.2	54.5 ± 8.8	53.8 ± 8.4	0.913
Body mass index, kg/m^2^	25.2 ± 2.8	25.7 ± 2.8	25.2 ± 2.7	25.7 ± 3.0	0.386
Male	122 (95.3%)	29 (90.6%)	64 (100.0%)	29 (90.6%)	0.043
Hypertension	49 (38.3%)	10 (31.2%)	28 (43.8%)	11 (34.4%)	0.430
Diabetes	12 (9.4%)	1 (3.1%)	8 (12.5%)	3 (9.4%)	0.332
Previous stroke	19 (14.8%)	4 (12.5%)	9 (14.1%)	6 (18.8%)	0.757
Paroxysmal AF	24 (18.8%)	8 (25.0%)	8 (12.5%)	8 (25.0%)	0.194
Prior RFCA	20 (15.6%)	4 (12.5%)	7 (10.9%)	9 (28.1%)	0.078
Hybrid (staged) RFCA	91 (71.1%)	21 (65.6%)	45 (70.3%)	25 (78.1%)	0.534
CHADS_2_ score	1.0 [0.0–2.0]	1.0 [0–1.0]	1.0 [0–2.0]	0.5 [0–2.0]	0.744
CHA_2_DS_2_ VASc score	1.0 [0.0–2.0]	1.0 [0–1.5]	1.0 [0–2.0]	1.0 [0–2.0]	0.846
NT-proBNP, pg/ml	254.8 [150.9–460.7]	211.3 [123.8–460.1]	265.1 [150.9–460.7]	280.7 [164.7–460.5]	0.732
Antiarrhythmic drugs	96 (75.0%)	26 (81.2%)	50 (78.1%)	20 (62.5%)	0.160
**Echocardiographic**					
LVEDD, mm	52.0 [49.0–54.5]	52.0 [49.0–56.0]	53.0 [49.5–55.0]	51.0 [49.0–53.0]	0.213
LVESD, mm	32.5 [30.0–35.0]	33.0 [29.0–36.5]	32.0 [30.0–35.0]	32.5 [30.0–35.0]	0.959
LVEF, %	60.0 [56.0–64.0]	60.5 [56.0–66.0]	60.0 [56.0–65.0]	57.5 [55.0–63.0]	0.199
*E*/*e*′	8.2 [6.3–10.1]	8.7 [6.4–10.1]	7.9 [6.3–10.2]	8.1 [6.3–9.9]	0.824
LAD, mm	45.0 [40.0–50.0]	45.0 [39.5–49.5]	46.0 [40.5–51.0]	43.5 [40.5–48.0]	0.462
LAVI, ml/m^2^	45.2 [36.4–54.8]	48.0 [38.0–51.6]	47.2 [37.5–55.5]	40.0 [32.6–53.4]	0.196
LA peak strain, %	15.3 [12.1–19.2]	18.7 [15.8–21.3]	15.5 [13.2–19.5]	10.6 [9.3–12.5]	<0.001
Stiffness index	0.5 [0.4–0.8]	0.5 [0.3–0.6]	0.5 [0.3–0.7]	0.8 [0.5–1.0]	0.002

Values are presented as mean ± SD, median [IQR], or *n* (%).

AF, atrial fibrillation; IQR, interquartile range; LA, left atrium; LAA, left atrial appendage; LAD, left atrial diameter; LAVI, left atrial volume index; LVEDD, left ventricular end-diastolic diameter; LVEF, left ventricular ejection fraction; LVESD, left ventricular end-systolic diameter; NOAC, novel oral anticoagulant; NT-proBNP, N-terminal-pro hormone B-type natriuretic peptide; RFCA, radiofrequency catheter ablation.

* Degree of LAA fibrosis was divided into 3 groups according to the IQR, as mild (1st quartile), moderate (2nd and 3rd quartile), or severe (4th quartile).

Patients were divided into 3 groups according to the interquartile range of degree of LAA fibrosis as mild (1st quartile), moderate (2nd and 3rd quartile), or severe (4th quartile). Peak LA strain and LA stiffness index were significantly different among the 3 groups, but other echocardiographic parameters including LA diameter and LAVI were not. Clinical variables were not significantly different among the 3 groups except for gender.

### Clinical outcome according to degree of LAA fibrosis

**Figure 3:**
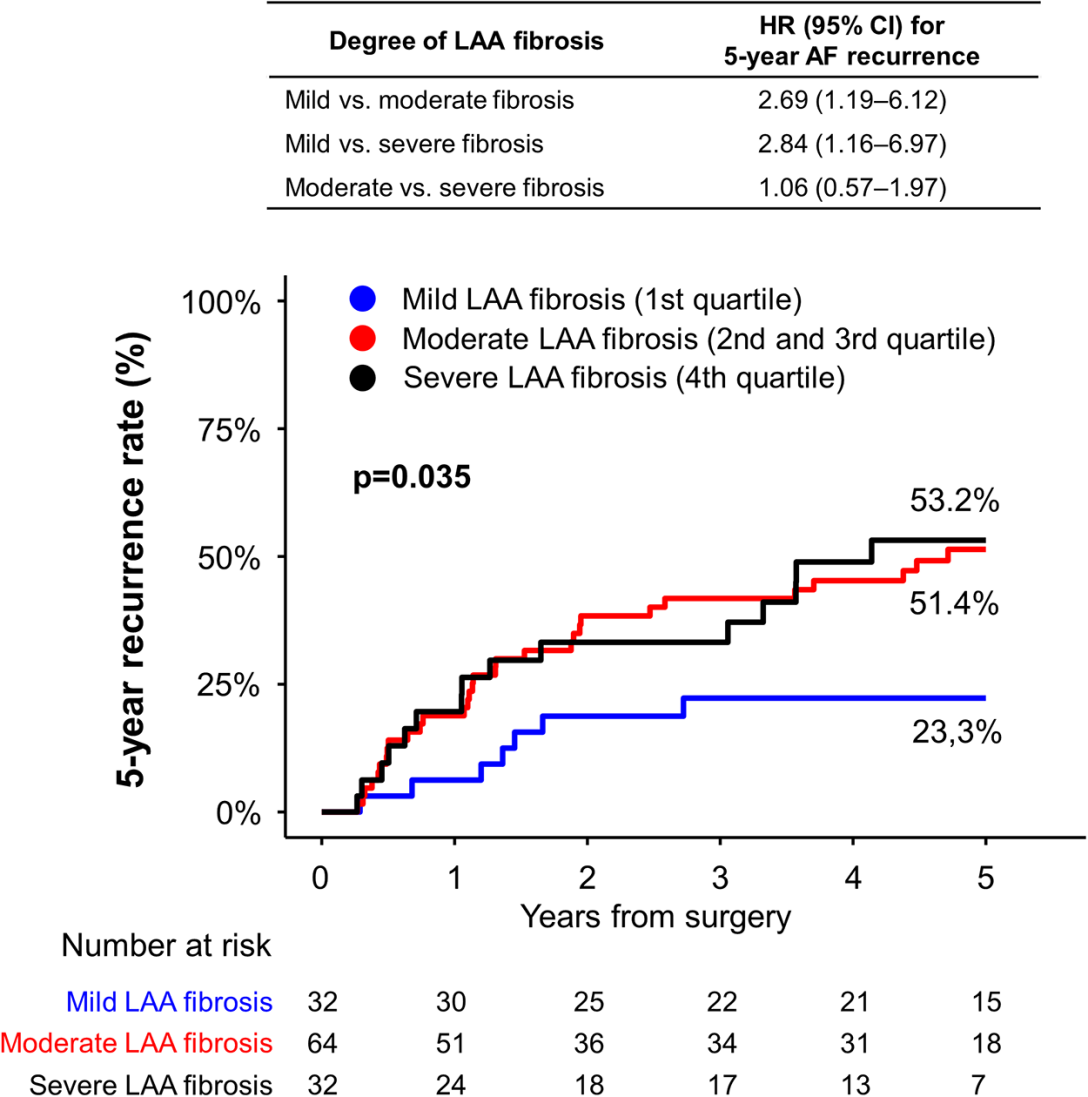
Five-year incidence of recurrent AF or AFL after thoracoscopic ablation according to degree of fibrosis. The degree of fibrosis was defined as mild (the first quartile), moderate (the second and third quartile), or severe (the fourth quartile). There was a significant difference in the risk of recurrent AF or AFL among the fibrosis group. AF, atrial fibrillation; AFL, atrial flutter; CI, confidence interval; HR, hazard ratio; LAA, left atrial appendage.

During the 5-year follow-up period, the risk of recurrent AF or AFL was significantly different according the 3 groups (log rank *p* = 0.035, Figure 3). The risk of 5-year AF or AFL recurrence was significantly lower in patients with mild LAA fibrosis (23.3%) compared with those with moderate (51.4%, hazard ratio [HR] 2.69; 95% confidence interval [CI] 1.19‒6.12; *p* = 0.018) and those with severe (53.2%; HR 2.84; 95% CI 1.16‒6.97; *p* = 0.023) LAA fibrosis.

The best cutoff value of degree of LAA fibrosis for the risk of 5-year AF or AFL recurrence was 33.6%, which was similar to the definition of mild LAA fibrosis (Supplementary Figure S2).

### Prevalence of mild LAA fibrosis according to baseline variables

**Figure 4:**
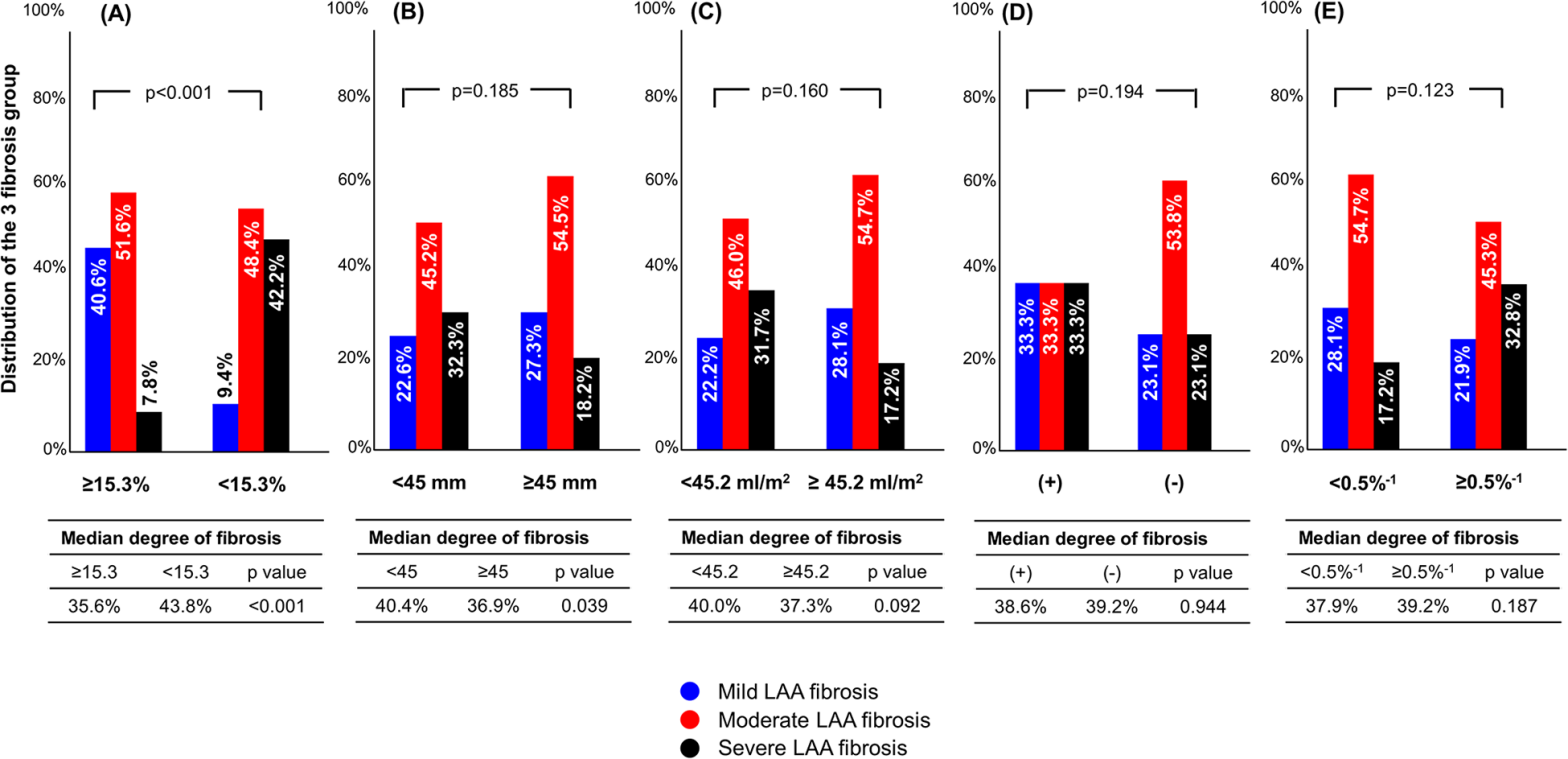
Distribution of fibrosis group according to preoperative parameters. The cutoff values were determined as the median of baseline parameters in overall population. (**A**) LA strain; (**B**), LA diameter, (**C**), LA volume index; (**D**), paroxysmal AF; (**E**), LA stiffness index. AF, atrial fibrillation; LA, left atrium.

Figure 4 shows distributions of the 3 fibrosis groups according to the median value of baseline variables. In patients with LA strain ≥15.3%, 40.6% of patients had mild LAA fibrosis, whereas only 9.4% had mild fibrosis in those with LA strain <15.3% (*p* < 0.001). The proportion of mild LAA fibrosis was not significantly different between LA diameter <45 vs. ≥45 mm, LAVI <45.2 vs. ≥45.2 ml/m^2^, paroxysmal AF vs. non-paroxysmal AF, or LA stiffness index <0.5%^−1^ vs. ≥0.5%^−1^.

### Independent predictor for mild LAA fibrosis

Lower LA strain was the only parameter significantly associated with mild LAA fibrosis in univariable and multivariable analysis (Table 2).

**Table 2:** Variables associated with mild fibrosis of left atrial appendage.

	Univariable		Multivariable	
	Coefficient	*p* value	Coefficient	*p* value
LA strain, %	0.10	0.005	0.14	0.002
Age, years	0.004	0.879	0.02	NS^*^
Female	1.17	0.167	1.79	NS
CHA2DS2 VASc	−0.12	0.511	−0.28	NS
Paroxysmal AF	0.51	0.299	−0.12	NS
NT-proBNP	−0.001	0.835	−0.0001	NS
LVESD, mm	0.02	0.678	0.02	NS
LAVI, ml/m^2^	−0.003	0.842	0.005	NS
*E*/*e*′	0.05	0.234	0.05	NS
LA diameter^**^	−0.01	0.750	NA	NA
Stiffness index^**^	−0.29	0.585	NA	NA

AF, atrial fibrillation; LA, left atrium; LAA, left atrial appendage; LAVI, left atrial volume index; LVESD, left ventricular end-systolic diameter; NA, not available; NT-proBNP, N-terminal-pro hormone B-type natriuretic peptide.

^*^ Not significant (NS) with a *p* value ≥0.05.

^**^ LA diameter and stiffness index were not included in multivariable analysis due to overlap.

**Figure 5:**
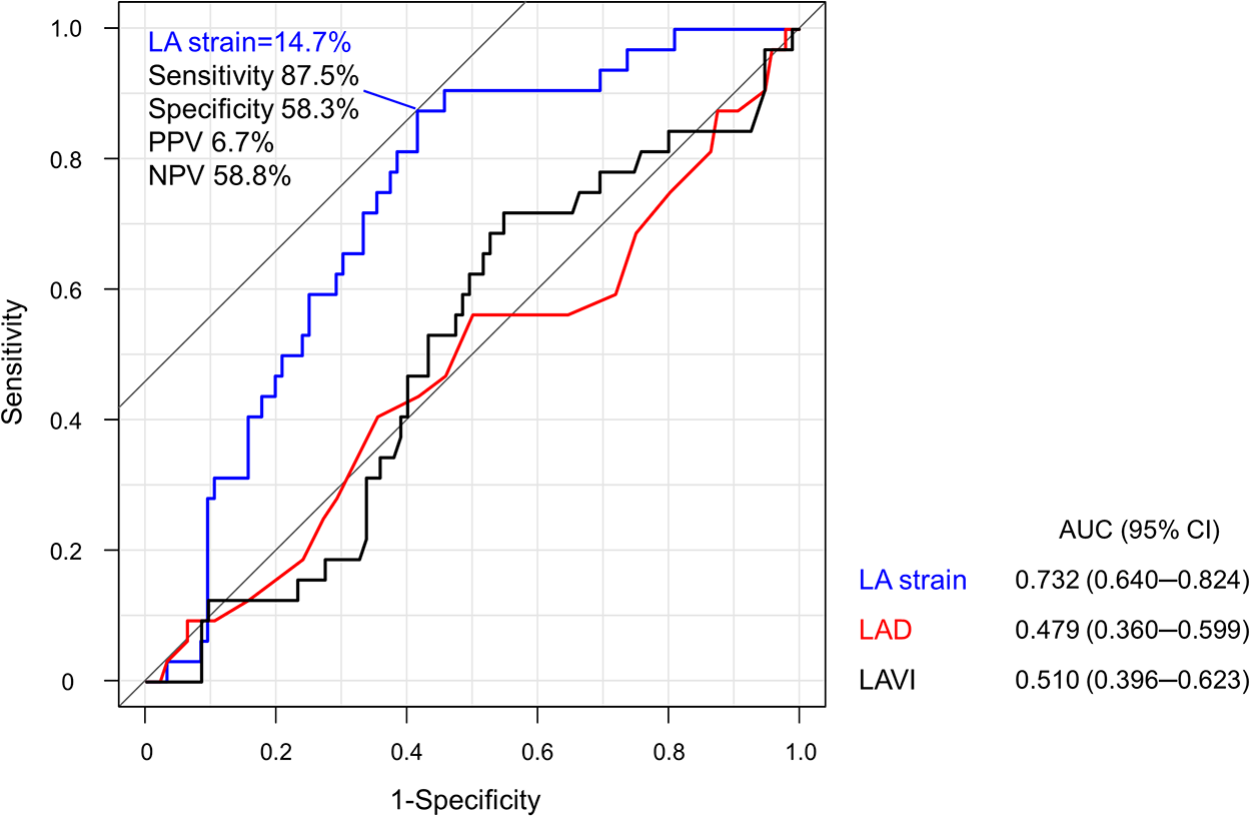
Receiver-operating characteristic analysis for the optimal cutoff value to predict mild LAA fibrosis. LA strain of 14.7% was the best cutoff value for predicting mild LAA fibrosis. LA, left atrium; LAD, left atrial diameter; LAVI, left atrial volume index; NPV, negative predictive value; PPV, positive predictive value.

The best cutoff value of LA strain for predicting mild LAA fibrosis was 14.7% with area under the curve of 0.732 (Figure 5). In patients with LA strain ≥14.7%, 40% of patients had mild LAA fibrosis, whereas only 6.9% had mild LAA fibrosis in those with LA strain >14.7% (*p* = 0.001).

### Subgroup analysis excluding patients with paroxysmal AF

A total of 104 patients with non-paroxysmal AF were included in a subgroup analysis, comprising 24 (23.1%) with mild LAA fibrosis, 56 (53.8%) with moderate LAA fibrosis, and 24 (23.1%) with severe LAA fibrosis. Significant differences were observed in LA strain and LA stiffness index among the three groups (Supplementary Table S1). Of the clinical and echocardiographic variables, only LA strain was significantly associated with mild LAA fibrosis (Supplementary Table S2).

## Discussion

The main findings of this study were as follows. First, mild LAA fibrosis was associated with a lower risk of recurrent AF or AFL at 5 years after TTA for AF. However, the risk of recurrent AF or AFL was not significantly different between the moderate and severe LAA fibrosis. Second, LA strain was the only parameter for predicting mild LAA fibrosis invasively assessed, whereas the conventional echocardiographic parameters such as LA diameter and LAVI were not.

Atrial fibrosis is a main pathophysiology that disrupts electrical activity and results in conduction abnormalities (3, 5, 22). In patients with AF, attempts to predict outcomes by quantifying atrial fibrosis have been largely based on MRI. Degree of fibrosis assessed by delayed enhancement MRI is known to be independently associated with arrhythmia recurrence after catheter ablation (9, 10, 23, 24). However, data on the prognosis of rhythm control management in patients with AF based on histologic evaluation are limited. In observational studies analyzing surgical specimens, advanced LA fibrosis was related with an unsuccessful maze operation (7) or a reduced recovery of atrial mechanical contraction (19). Recently, TTA has been highlighted with minimal invasiveness and favorable outcomes (25–27) because traditional maze procedure is highly invasive in patients not planned for cardiac surgery. Ma et al. reported that the degree of LAA fibrosis was an independent predictor of 5-year recurrence rate after TTA (28). In our study, only mild LAA fibrosis was significantly associated with a lower recurrence rate compared with severe fibrosis, but moderate fibrosis was not. The different findings between the 2 TTA studies may be due to patients' characteristics. Our study patients had more advanced AF, such as larger LA diameter, higher prevalence of persistent AF and history of previous catheter ablation, and higher recurrence rate compared with the study by Ma et al. Our finding suggests that more complex or enhanced therapeutic strategies may be needed to prevent AF recurrence in patients with advanced atrial fibrosis (10).

Limited data are available on the relationship between echocardiographic parameters and degree of fibrosis determined by histopathology in patients with AF. In this issue, LA strain has emerged as a potential parameter representing atrial function and remodeling. In patients undergoing mitral valve surgery, LA strain was independently correlated with the degree of LA fibrosis (13, 29). In these studies, however, all patients had either severe mitral stenosis or mitral regurgitation resulting in chronic pressure or volume overload on LA, and a large number of patients had no AF. A recent study reported that LA strain had a good correlation with degree of LA fibrosis, but the results were derived from patients with advanced heart failure undergoing heart transplantation (14). In our study, LA strain was an independent predictor of mild LAA fibrosis that was related with favorable outcome after TTA. Patients with LA strain ≥15.3%, comped with those with LA strain <15.3%, had a 4-fold higher prevalence of mild LAA fibrosis. Whereas classic indices such as LA diameter, LAVI, *E*/*e*′, or paroxysmal AF were not associated with the degree of LAA fibrosis. In line with previous studies, our study suggests that in patients with lone AF, LA fibrosis cannot be predicted by classic morphologic parameters, while LA strain predicts the atrial fibrosis and provides prognostic information. However, it should be noted that these findings were derived from patients undergoing TTA Therefore, it is necessary to verify the clinical implications of LA strain in a wider population of individuals with AF. The absence of a significant difference in fibrosis degree between patients with lower and higher LA stiffness index may be attributed to the lack of a significant difference in *E*/*e*′ values among the mild, moderate, and severe fibrosis groups. This could have resulted in a dilution of the difference in LA strain, leading to our observed findings.

Several factors may influence LA strain measurements, such as the patient's rhythm status during measurements or prior radiofrequency catheter ablation. However, we observed a consistent association between higher LA strain and less LAA fibrosis after excluding patients with paroxysmal AF. Given that one of the indications of thoracoscopic surgical ablation is for patients who have failed percutaneous AF ablation (30), a history of prior radiofrequency ablation is relatively common in TTA candidates. In a meta-analysis of TTA, 52.9% of patients had a history of one or more failed catheter ablation (31). Therefore, the prevalence of prior radiofrequency catheter ablation of 15.6% in our study is not particularly high for use of LA strain in this population. In fact, out of 108 patients without previous RFCA, those with mild, moderate, and severe LAA fibrosis had median LA strain values of 18.7% [15.8–20.9], 15.6% [13.1–19.2], and 10.7% [9.6–13.8], respectively (*p* < 0.001).

Whether the LAA fibrosis is representative of LA fibrosis is a separate issue. A previous autopsy study found similar degrees of fibrosis in different locations of the LA in patients with AF (32). Moreover, a study using high-density bipolar voltage mapping suggested that voltage reduction in the LA is a diffuse process associated with fibrosis, indicating that AF-related structural remodeling is widespread (33). Our study revealed a significant association between mild LAA fibrosis and a reduced risk of AF recurrence after TTA, which could be predicted by LA strain, a surrogate for LA remodeling. These findings suggest that LAA fibrosis may serve as a marker for whole LA remodeling in patients with AF, which is consistent with previous studies.

Our study had several limitations. First, this was a single-center retrospective study. Characteristics of patients and experience with TTA might be different by centers. Second, the best cutoff value or predictive power of LA strain might be different by image quality of echocardiography, experience for delineation of region of interests, or speckle-tracking software (21). Third, the present findings were derived from limited population who underwent TTA. However, the relationship between preoperative LA strain and degree of fibrosis would not be influenced by treatment modality. In addition, although data are limited, studies on Maze procedure also have suggested the degree of atrial fibrosis as a prognostic factor after AF ablation. Last, as this study was observational, it may not have had sufficient statistical power to evaluate the predictive value of several echocardiographic variables, such as LA diameter and LA volume index, on LAA fibrosis. However, our findings suggest that patients with LA enlargement did not have more fibrosis than those without LA enlargement, either numerically or statistically.

The strength of our study is that it directly compared noninvasive parameters with histologically confirmed atrial fibrosis in patients with AF and provides prognostic implications of atrial fibrosis and LA strain in rhythm control management. Future large-scale studies are needed to confirm the current findings.

## Conclusions

In patients with AF, mild LAA fibrosis was associated with a lower risk of 5-year AF recurrence after TTA. LA strain was the only predictor of mild LAA fibrosis that reflects a lower risk of 5-year AF recurrence.

## Data Availability

The raw data supporting the conclusions of this article will be made available from the corresponding author [DSJ], upon reasonable request.
