# Supplementary material for: Left atrial strain predicts fibrosis of left atrial appendage in patients with atrial fibrillation undergoing totally thoracoscopic ablation
Source: Front Cardiovasc Med. 2023 May 17;10:1130372. doi: 10.3389/fcvm.2023.1130372 (PMC10229857; doi:10.3389/fcvm.2023.1130372)
Supplement: Supplementary file 1 [file Table1.docx]

**Left atrial strain predicts fibrosis of left atrial appendage in patients with atrial fibrillation undergoing totally thoracoscopic ablation**

Table of contents

- Supplement Tables
- Supplement Figures

**Supplement Table 1. Baseline characteristics of patients with non-paroxysmal AF**

| **Variables** | **Overall (n=104)** | **Mild fibrosis***  **(<33.1%, n=24)** | **Moderate fibrosis**  **(33.1–44.7%, n=56)** | **Severe fibrosis**  **(≥44.7% n=24)** | **p value** |
| --- | --- | --- | --- | --- | --- |
| LAA fibrosis, % | 38.6 [34.1–44.0] | 29.4 [27.4–31.3] | 38.6 [36.1–40.6] | 52.2 [47.6–55.2] | <0.001 |
| Clinical |  |  |  |  |  |
| Age, years | 54.4 ± 8.5 | 55.2 ± 7.6 | 54.4 ± 9.2 | 53.6 ± 8.0 | 0.797 |
| Body mass index, kg/m^2^ | 25.5 ± 2.7 | 26.3 ± 2.6 | 25.4 ± 2.7 | 24.9 ± 2.5 | 0.179 |
| Male | 100 (96.2%) | 22 (91.7%) | 56 (100.0%) | 22 (91.7%) | 0.088 |
| Hypertension | 45 (43.3%) | 9 (37.5%) | 28 (50.0%) | 8 (33.3%) | 0.313 |
| Diabetes | 8 (7.7%) | 1 (4.2%) | 7 (12.5%) | 0 (0%) | 0.120 |
| Previous stroke | 13 (12.5%) | 2 (8.3%) | 7 (12.5%) | 4 (16.7%) | 0.683 |
| Prior RFCA | 12 (11.5%) | 4 (16.7%) | 5 (8.9%) | 3 (12.5%) | 0.602 |
| Hybrid (staged) RFCA | 76 (73.1%) | 16 (66.7%) | 40 (71.4%) | 20 (83.3%) | 0.394 |
| CHADS_2_ score | 1.0 [0.0–1.0] | 1.0 [0–1.0] | 1.0 [0–2.0] | 0 [0–1.5] | 0.441 |
| CHA_2_DS_2_ VASc score | 1.0 [0.0–1.5] | 1.0 [0–1.0] | 1.0 [0–2.0] | 0 [0–1.5] | 0.460 |
| NT-proBNP, pg/ml | 280.6 [175.7–482.1] | 225.2 [123.8–52.0] | 269.8 [165.7–464.9] | 317.2 [233.3–485.8] | 0.518 |
| Antiarrhythmic drugs | 77 (74.0%) | 20 (83.3%) | 42 (75.0%) | 15 (62.5%) | 0.251 |
| Echocardiographic |  |  |  |  |  |
| LVEDD, mm | 52.0 [50.0–54.5] | 54.0 [49.5–56.5] | 53.0 [50.0–55.0] | 50.5 [49.5–52.5] | 0.084 |
| LVESD, mm | 33.0 [30.0–35.0] | 33.0 [30.0–37.0] | 32.5 [30.5–35.0] | 31.5 [30.0–34.0] | 0.680 |
| LVEF, % | 59.5 [56.0–64.0] | 60.0 [56.0–64.0] | 59.0 [56.0–65.0] | 59.0 [55.5–63.0] | 0.682 |
| E/e’ | 8.1 [6.3–10.0] | 9.1 [7.3–10.6] | 7.7 [6.3–9.9] | 7.6 [6.2–9.8] | 0.186 |
| LAD, mm | 46.0 [42.0–51.0] | 48.0 [41.5–50.5] | 47.0 [42.0–51.0] | 44.0 [41.5–51.5] | 0.749 |
| LAVI, ml/m^2^ | 48.2 [37.8–55.5] | 50.2 [43.4–53.5] | 48.2 [40.2–57.7] | 40.5 [33.3–56.5] | 0.318 |
| LA peak strain, % | 14.8 [12.0–18.1] | 17.4 [15.3–20.7] | 14.8 [13.0–18.6] | 10.5 [9.3–12.2] | <0.001 |
| Stiffness index | 0.6 [0.4–0.8] | 0.5 [0.4–0.7] | 0.5 [0.4–0.8] | 0.8 [0.5–1.0] | 0.031 |

Values are presented as mean ± SD, median [IQR], or n (%).

Abbreviations: AF, atrial fibrillation; IQR, interquartile range; LA, left atrium; LAA, left atrial appendage; LAD, left atrial diameter; LAVI, left atrial volume index; LVEDD, left ventricular end-diastolic diameter; LVEF, left ventricular ejection fraction; LVESD, left ventricular end-systolic diameter; NOAC, novel oral anticoagulant; NT-proBNP, N-terminal-pro hormone B-type natriuretic peptide; RFCA, radiofrequency catheter ablation.

**Supplement Table 2. Variables associated with mild fibrosis of left atrial appendage**

|  | **Univariable** | | **Multivariable** | |
| --- | --- | --- | --- | --- |
|  | **Coefficient** | **p value** | **Coefficient** | **p value** |
| LA strain, % | 0.13 | 0.007 | 0.20 | 0.001 |
| Age, years | 0.02 | 0.568 | 0.04 | NS^*^ |
| Female | 1.27 | 0.219 | 2.06 | NS |
| CHA2DS2 VASc | -0.11 | 0.606 | -0.37 | NS |
| NT-proBNP | -0.0002 | 0.802 | 0.0006 | NS |
| LVESD, mm | 0.06 | 0.236 | 0.03 | NS |
| LAVI, ml/m^2^ | 0.003 | 0.854 | -0.007 | NS |
| E/e’ | 0.11 | 0.107 | 0.12 | NS |
| LA diameter^**^ | 0.02 | 0.540 | NA | NA |
| Stiffness index^**^ | 0.25 | 0.631 | NA | NA |

* Not significant (NS) with a p value ≥0.05

** LA diameter and stiffness index were not included in multivariable analysis due to overlap.

Abbreviations: AF, atrial fibrillation; LA, left atrium; LAA, left atrial appendage; LAVI, left atrial volume index; LVESD, left ventricular end-systolic diameter; NA, not available; NT-proBNP, N-terminal-pro hormone B-type natriuretic peptide.

**Supplement figure 1. Measurement of left atrial strain**


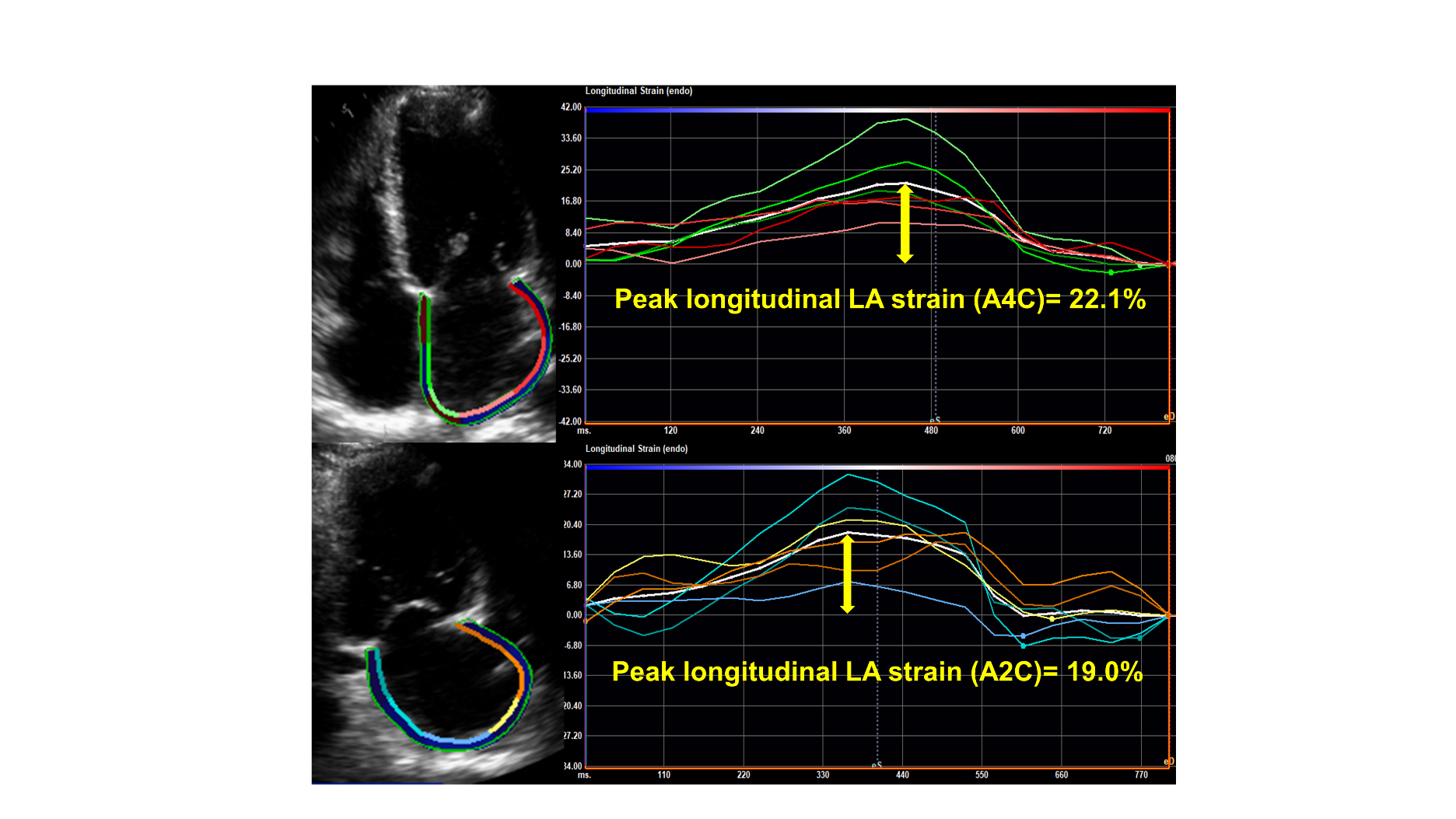


Abbreviations: A2C, apical 2 chamber view; A4C, apical 4 chamber view

**Supplement figure 2. Detecting the best cutoff value of left atrial fibrosis**

**
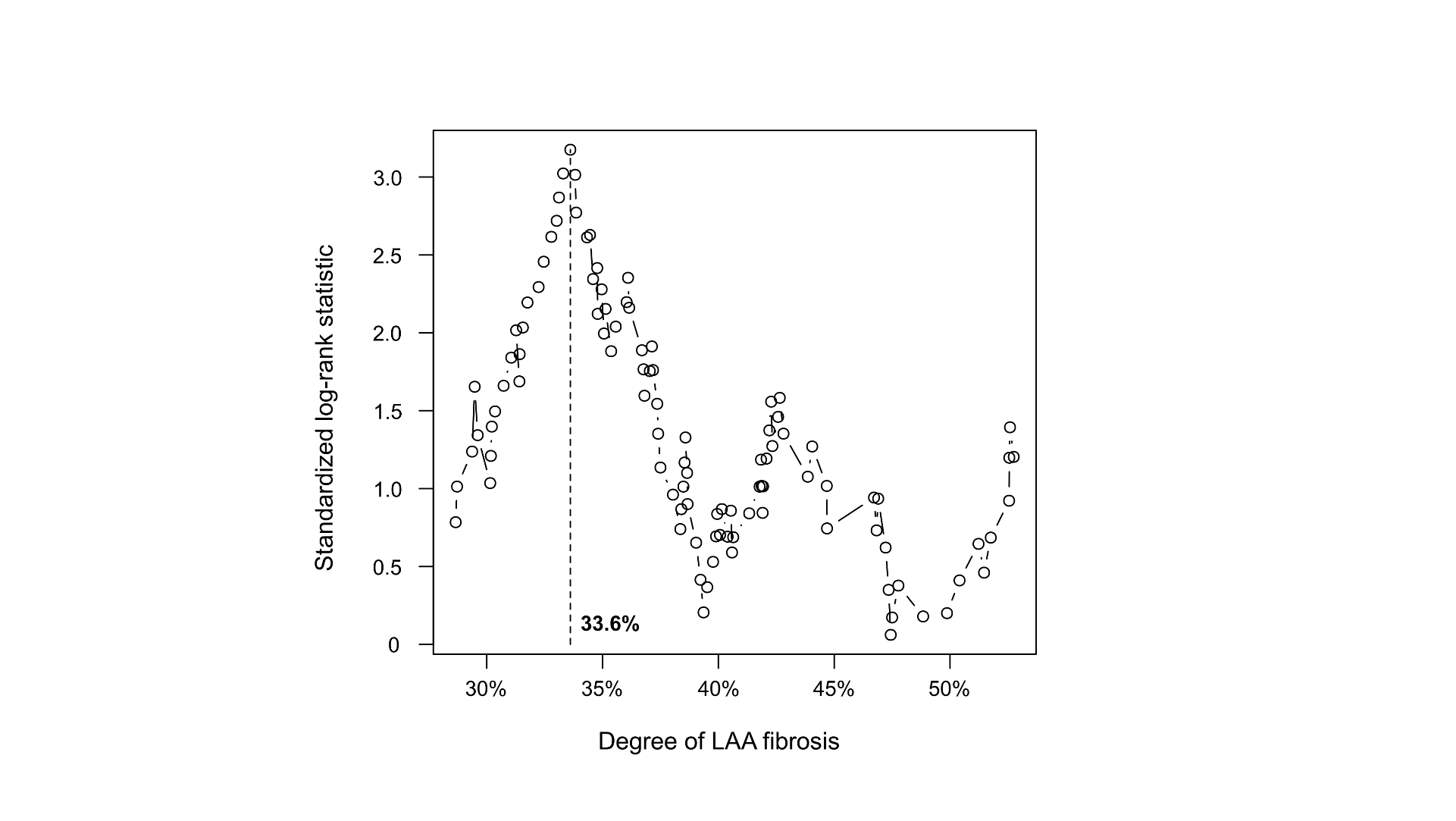
**

Abbreviations: LAA, left atrial appendage.
